# NTP Multigenerational Study of Environmental Estrogens

**DOI:** 10.1289/ehp.114-a348

**Published:** 2006-06

**Authors:** Julia R. Barrett

Nearly 10 years ago, researchers with the National Toxicology Program (NTP) and the National Center for Toxicological Research (NCTR) began a complex set of experiments in rats to determine whether exposure to estrogenic compounds throughout life and across generations could cause changes in development or patterns of endocrine-related cancers at doses that cause only subtle reproductive effects. Now, at last, specialists in the field of endocrine-active chemicals are close to getting a first look at the findings from these studies.

The three compounds chosen for study—genistein, ethinyl estradiol, and *p*-nonylphenol—represent a natural estrogenic substance, a drug, and an industrial chemical, respectively. The first experiments for all three compounds focused on determining appropriate dose ranges for later multigenerational studies. Additionally, studies were conducted with genistein and ethinyl estradiol to determine whether and how the carcinogenic potential of these substances changed across generations following long-term chronic exposure.

On 12 June 2006, the first reports based on these experiments will undergo peer review at a one-day meeting at the NIEHS, with final publication expected later this year and additional reports scheduled for review in 2007. The reports to be reviewed on June 12 center on genistein, an estrogen-like compound found in soy, and detail the results of dose range–finding studies and multigenerational reproductive and carcinogenesis experiments.

## Years in the Making

According to John Bucher, deputy director of the NIEHS Environmental Toxicology Program and a member of the group that designed and monitored the studies, the potential for endocrine disruption affecting development has been a topic of interest at the NIEHS since the late 1970s, when the institute held its first conference to examine the matter. Through the 1980s and into the 1990s, accumulating research established solid biological plausibility for the idea that small perturbations in hormonal status triggered by environmental exposures could ultimately affect development.

There were still many unknowns, however, according to Robert Chapin, a former NIEHS reproductive toxicologist now at Pfizer. “As is most often the case in science,” he says, “there was a whole lot more that was unknown than was known about low-dose exposure to estrogenically active chemicals. There were lots of claims being made about these [chemicals] that were not biologically plausible.”

Following the 1994 NIEHS-sponsored meeting “Estrogens in the Environment III,” Bucher, Suzanne Snedeker (a former NIEHS scientist now at Cornell University), Chapin, and others at the NTP decided to put together a new series of experiments. The goal: to evaluate the potential of estrogenic influences during development to change developmental patterns for sexually related characteristics or hormonally mediated tumor patterns in animals as they aged. “We thought the NTP would be able to do this in a way that few other groups could, in a very comprehensive and thorough manner,” says Bucher.

The NCTR’s expertise in such large-scale studies and its interest in the area made it the ideal partner. This branch of the FDA conducts toxicological research to determine the human exposure to and risks of products that are regulated by that agency. With that, the NTP entered into an interagency study with K. Barry Delclos, the principal investigator at the NCTR, and Retha R. Newbold, the principal investigator at the NIEHS.

## Selecting the Candidates

The researchers originally selected five chemicals for study: methoxychlor, genistein, ethinyl estradiol, *p*-nonylphenol, and vinclozolin. The first four seemed to have estrogenic properties in addition to other, unique characteristics, and their inclusion was expected to provide the opportunity to tease out which effects could be related to estrogenicity versus the responses specific to the individual chemical.

After dose range–finding studies were completed in 2001, the researchers decided against conducting multigenerational studies on methoxychlor and vinclozolin. There were several reasons for this decision, including the fact that methoxychlor didn’t exhibit enough of an estrogenic effect to justify doing the additional studies, and that vinclozolin was the only antiandrogen, with no comparison compounds being tested.

The doses of 5, 100, and 500 milligrams of genistein per kilogram per day were selected very carefully. “What we were interested in was studying a wide range of concentrations,” says Bucher. “We wanted to select a top dose for the multigenerational studies that had a clear biological effect but didn't affect the animals to the extent that reproduction would be inhibited. We wanted to put the lower doses in the range of human exposures.”

## Studies Begin

Exposure for the parental (F_0_) generation began when the animals were weaned to feed supplemented with the test compound. The feed did not contain alfalfa or soy, because both contain naturally occurring estrogenic compounds. The subsequent F_1_, F_2_, and F_3_ generations of offspring experienced exposure to the chemicals prenatally but much less so through lactation; subsets of the F_1_ and F_2_ generations consumed supplemented feed upon weaning, but exposure ceased for the F_3_ generation at weaning. The F_4_ generation was unexposed. Subsets of animals were examined at each stage of development, and additional subsets of the F_1_ and F_3_ generations were used for the chronic exposure assays. “It was a long, complicated series of studies,” says Bucher.

The complexity was necessary, however, because a major thrust of the studies was to test whether effects worsened over succeeding generations and whether they were reversible if exposure ended. Chapin explains, “From the public policy exposure–decision point of view, if you could show that things started to get better once you stopped exposure, that would mean that . . . if we did something about it, it would reverse any endocrine disruptor–mediated reproductive compromise that might be happening in human populations.”

The studies could have been even more complex, according to Delclos. “I can think of a few things off the top of my head that would have been nice to do if resources were unlimited,” he says. He speculates that including more dose groups would have allowed for better characterization of the dose–response curve, especially in the lower-dose region reflecting likely human exposure levels. Additionally, no one rodent model is ideal. Although the rats used in these experiments are well suited for multigenerational studies and have low background rates of some reproductive tumors, they do have high background rates of pituitary tumors and, in females, mammary tumors. This makes it more difficult to pick up on subtle changes that might be occurring.

With regard to genistein, Delclos indicates that the use of dosed feed led to very low exposure during the early neonatal period because the transfer of genistein through the milk was minimal; thus, the exposure was too low to have an effect. Overall, comparison of the pure compound with more complex soy extracts would have been of interest, to provide a better sense of the effects of real-life exposure.

## Food for Thought

Nevertheless, the studies have yielded in-depth information on how mammals respond across developmental stages and through several generations to estrogenic compounds at exposures relevant to human health. Findings from different sets of experiments may ultimately be compared between test substances to elucidate common responses to estrogenic compounds.

These studies are a classic example of the value of the NTP, says Chapin. “They’re much larger than any academic or even most contract research organizations would be able to accomplish, [and] they are clearly in the public interest,” he says. “It’s a perfect reason why we have an organization that does this kind of stuff—to do the kind of studies that are critically important for public health but don’t get done anywhere else.”

## Figures and Tables

**Figure:**
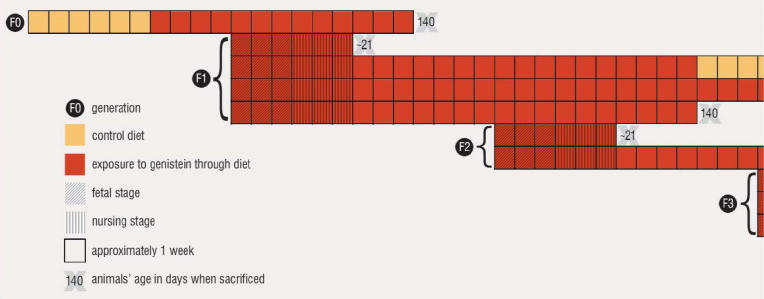

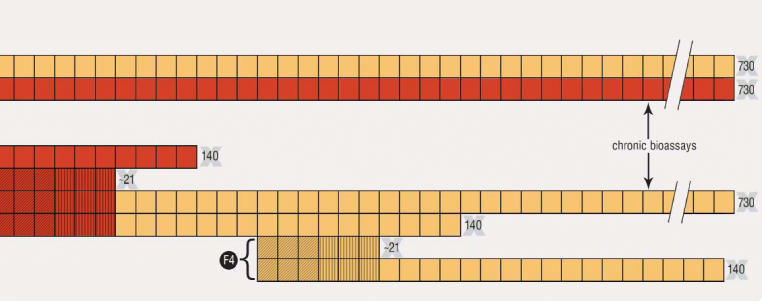
Multigenerational Rodent Study: Genistein Dosing Schedule

